# A systematic scoping review of faropenem and other oral penems: treatment of Enterobacterales infections, development of resistance and cross-resistance to carbapenems

**DOI:** 10.1093/jacamr/dlac125

**Published:** 2022-12-22

**Authors:** Sumanth Gandra, Satoshi Takahashi, Fanny S Mitrani-Gold, Aruni Mulgirigama, Diogo A Ferrinho

**Affiliations:** Division of Infectious Diseases, Washington University School of Medicine, St. Louis, MO, USA; Division of Laboratory Medicine, Sapporo Medical University Hospital, Sapporo, Japan; Department of Infection Control and Laboratory Medicine, Sapporo Medical University School of Medicine, Sapporo, Japan; Epidemiology, GSK, Collegeville, PA, USA; Global Medical Affairs, GSK, Brentford, Middlesex, UK; Global Medical Affairs, GSK, Brentford, Middlesex, UK

## Abstract

**Background:**

Antimicrobial resistance is an urgent global healthcare concern. Beyond carbapenems as broad-spectrum, often ‘last resort’ antibiotics, oral penem antibiotics currently are approved only in Japan and India, used for the treatment of indications including urinary tract infections (UTIs). Exploring oral penem use to better understand the impact of antibiotic resistance on public health would help inform the management of infectious diseases, including UTIs.

**Scoping Review Methodology:**

This scoping review investigated the impact of faropenem and other oral penems on Enterobacterales infection treatment and evaluated evidence for faropenem resistance and cross-resistance to carbapenems. PubMed, Embase, J-STAGE and CiNii were searched for relevant English- or Japanese-language articles published between 1 January 1996 and 6 August 2021.

**Key Findings:**

From 705 unique publications, 29 eligible articles were included (16 *in vitro* studies; 10 clinical trials; 2 *in vitro* and *in vivo* studies; and 1 retrospective medical chart review). Limited evidence described faropenem to treat infectious disease; only four randomized clinical trials were identified. Faropenem dosing regimens varied broadly within and between indications. One study indicated potential dependence of penem efficacy on underlying antibiotic resistance mechanisms, while several studies reported UTI persistence or recurrence after faropenem treatment. *In vitro* MIC data suggested some potential bacterial resistance to faropenem, while limited clinical data showed resistance emergence after faropenem treatment. Preliminary *in vitro* evidence suggested faropenem resistance might foster cross-resistance to carbapenems. Overall, very limited clinical evidence describes faropenem for treating infectious diseases. Preclinical and clinical research investment and dedicated community surveillance monitoring is crucial for understanding faropenem treatment patterns, resistance and potential cross-resistance to carbapenems.

## Introduction

Antimicrobial resistance is a huge challenge for the effective prevention and treatment of infectious diseases worldwide.^1,2^ Over time, infectious agents such as bacteria, viruses and fungi acquire resistance to anti-infectives, which is associated with disease progression, increased numbers of treatment cycles and hospital stays, negative impacts on health-related quality of life, and higher patient mortality.^1,3,4^ Rates of antimicrobial resistance are driven primarily by the excessive and/or inappropriate use of antimicrobial agents, with significant implications for increased healthcare costs,^5^ suboptimal patient care^6^ and an increased risk of adverse effects.^7^

A range of antimicrobial agents with various mechanisms of action can be used to treat infectious diseases. These agents mostly comprise inhibitors of protein or cell wall synthesis, such as aminoglycosides (e.g. gentamicin, streptomycin) β-lactams (e.g. penicillins, cephalosporins), macrolides (e.g. azithromycin) and tetracyclines (e.g. doxycycline), but also include inhibitors of folic acid synthesis (e.g. sulphonamides, diaminopyrimidines), topoisomerase inhibitors (e.g. fluoroquinolones), cell membrane disruptors (e.g. polymyxins) and inhibitors of RNA synthesis (e.g. rifamycins).^8^

Carbapenems and penems are two classes of broad-spectrum β-lactam antibiotics. Their antimicrobial activity is derived through binding to and inhibition of PBPs, which ultimately inhibits the synthesis of the bacterial cell wall component peptidoglycan.^8,9^ Carbapenems were originally isolated as natural products produced by some bacterial species and have a carbon atom at position C-1 in the β-lactam ring; penems, by contrast, do not occur naturally and are chemically synthesized, possessing a sulphur atom at the C-1 position of the β-lactam ring.^9–11^ Longer C–S bond lengths and smaller C–S–C bond angles alter the 5-membered ring conformation in oral penems versus carbapenems, leading to reduced intra-ring stress.^12^ Faropenem, unlike carbapenems, also lacks a protonatable C-2 side chain; the remarkable chemical stability of penems may be underscored by conformational restraints acting on the C-2 cyclic tetrahydrofuran ring. While faropenem demonstrates high oral bioavailability (around 70%–80% in its ester prodrug form), carbapenems must be administered parenterally. Efforts to improve the oral bioavailability of carbapenems are ongoing.^9,13^

Several hundred β-lactam antibiotics exist, but the carbapenems have the broadest spectrum of activity and greatest potency against Gram-positive and Gram-negative bacterial species.^9^ For this reason, when treatment with other antibiotics fails, carbapenems are used as the last-line antibiotics for treating severe and/or resistant bacterial infections^9^ that often are associated with high morbidity and mortality. Critically, however, bacterial resistance to carbapenems is increasing worldwide.^9,14–17^ Carbapenem resistance may arise via several mechanisms but most often is the result of breakdown by β-lactamases, including the carbapenem-specific Class A and Class D carbapenemases (such as KPC enzymes and OXA-23, respectively).^9,18,19^ Other resistance mechanisms include removal via efflux pumps and expression- or function-altering mutations to porins and PBPs. Accumulation of carbapenem resistance mechanisms may also occur under antibiotic selective pressure.^20^ Patterns of resistance to carbapenems, and antibiotics in general, reflect the influence of ecological and other factors.^14,21–23^ It is vitally important to preserve carbapenems as their misuse to treat non-severe infections when other treatment options are available could threaten their value as ‘last resort’ agents.

Carbapenems as IV formulations are available in most countries for the treatment of severe, complicated and/or resistant bacterial infections, including those affecting the respiratory, abdominal and urinary tracts, and the skin.^24^ An oral form of the carbapenem tebipenem is licensed for use in Japan in paediatric patients with serious respiratory infections and is being developed for treating adult infections.^25–27^ The US FDA recently issued a Complete Response Letter to a new drug application for oral tebipenem for the treatment of complicated urinary tract infections (cUTIs), concluding that data submitted from the Phase 3 cUTI study were insufficient to support approval and that further clinical development was required.^28^ An oral penem, faropenem, is available in Japan and India for the treatment of urinary tract infections (UTIs), respiratory tract infections, skin and skin structure infections and gynaecological infections.^29,30^ Faropenem is available in Japan as 150 and 200 mg tablets and the recommended faropenem regimen for treating uncomplicated UTIs (uUTIs) is 600 mg daily for 7 days.^31^ The 2019 standard treatment guidelines from the Indian Government do not list faropenem for treating UTI,^32^ although Medindia recommends 200–300 mg of faropenem twice daily for UTIs,^33^ and dosing formulations available in India include 150 and 200 mg.^34^ In Japan, faropenem is not prescribed as a first-line treatment and its use is restricted to specific clinical scenarios, including the treatment of infections with ESBL-producing bacteria.^31^ The penem antibiotic sulopenem has recently been reviewed^35^ and an oral formulation is currently in clinical development for treating a variety of infections,^36^ although the US FDA has requested additional data to support its approval for the treatment of uUTIs.^37^ The continued development of oral penem formulations could potentially increase the use of sulopenem and other penems for treating infections in community and hospital settings.

Despite the background of carbapenem resistance, the public health impact of the structurally related class of oral penems such as faropenem remains poorly understood. Indeed, there is concern that increased use and inappropriate or off-label use of antibiotics like faropenem could accelerate cross-class resistance to carbapenems.^38^ This is especially concerning as faropenem consumption is rapidly increasing in India, a country already with a high prevalence of carbapenem-resistant Gram-negative bacterial infections.^39^ In India, faropenem sales increased by 154% between 2010 and 2014 (7.4 million standard units and 18.9 million standard units, respectively),^29^ and continued in a similar trend between 2018 and 2020 (approximately 1.75 million standard units and 3.25 million standard units, respectively).^40^ Moreover, susceptibility testing against faropenem is not routinely performed in clinical microbiology laboratories in India or Japan due to the absence of interpretative breakpoints for faropenem in guidelines issued by CLSI, EUCAST and the Japanese Society of Chemotherapy (JSC). Thus, there is an unmet need to explore the use of faropenem and oral penems and their impact on patterns of resistance to help inform patient management and improve antimicrobial stewardship and the treatment of infectious diseases, including UTIs.

We conducted a scoping literature review to evaluate the impact of faropenem on the treatment of Enterobacterales infections in the inpatient, outpatient and preclinical settings; and furthermore, to discuss the potential impact of faropenem use on the development of faropenem resistance and cross-resistance to carbapenem antibiotics, and what this might mean for the treatment of uUTIs in the community.

## Methods

### Research questions

Scoping reviews are a type of literature review that follow a systematic approach to identify main concepts, key themes and knowledge gaps on a particular topic,^41,42^ and they often have one or more relatively broad research questions. We conducted a scoping review of the available literature on faropenem in an attempt to answer three research questions: (1) What is the prevalence of faropenem resistance among Enterobacterales?; (2) Is there evidence for the emergence/development of faropenem resistance after drug exposure?; and (3) Is there evidence that development of resistance to faropenem leads to cross-resistance to non-oral carbapenems in Enterobacterales?

**Figure 1.**
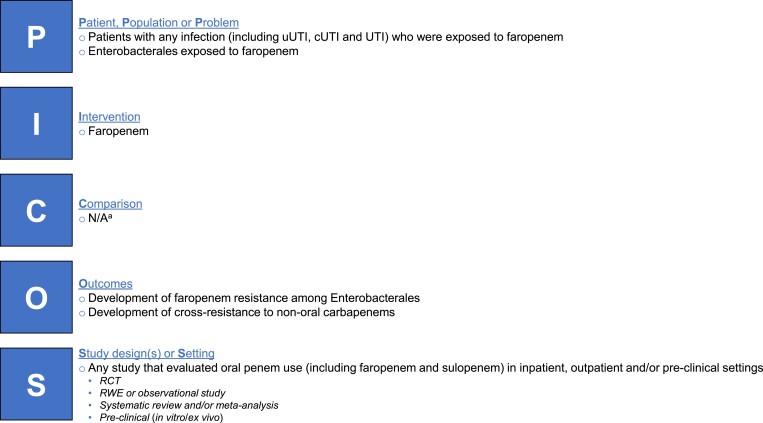
Summary of PICOS criteria for systematic scoping literature review. ^a^Studies of faropenem and comparators were included, but the scoping review did not specifically aim to compare faropenem with other treatments. N/A, not applicable; RCT, randomized controlled trial; RWE, real-world evidence.

We followed the Population, Intervention, Comparison, Outcomes and Study (PICOS) approach to identify eligible publications for review. Briefly, this included studies of patients with any infectious disease, or any Enterobacterales, that evaluated oral penem (faropenem) use and patterns of antibiotic resistance or cross-resistance to non-oral carbapenems, in inpatient, outpatient and/or preclinical settings. The PICOS criteria used for this review are summarized in Figure 1.

### Search strategy

The search strategy comprised electronic searches of the published literature using the PubMed (peer-reviewed manuscript records), Embase (peer-reviewed manuscript records and congress abstracts), J-STAGE (full-text Japanese articles) and CiNii (Japanese-language academic articles) databases, as well as manual searches of relevant congress proceedings where historical abstract information was available online (including congresses of the American Society for Microbiology, European Congress of Clinical Microbiology and Infectious Diseases, IDSA and the International Society of Infectious Diseases congresses, and those in Asia focusing on infectious diseases).

**Figure 2.**
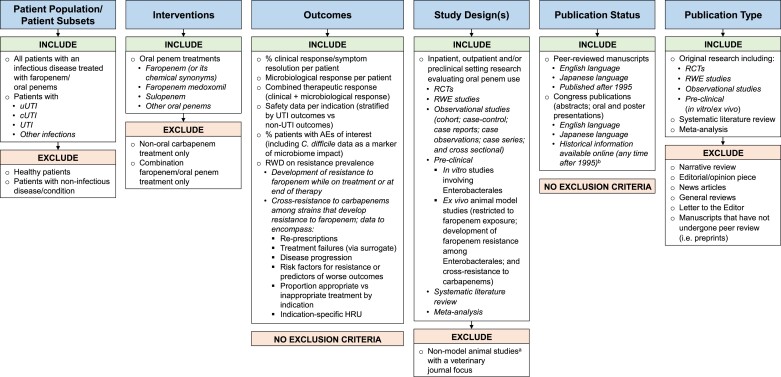
Inclusion and exclusion criteria for article title and abstract screen. ^a^Animals other than rats or mice. ^b^Online congress records searched manually for relevant publications. *C. difficile*, *Clostridioides difficile*; HRU, healthcare resource utilization; RCT, randomized controlled trial; RWD, real-world data; RWE, real-world evidence.

The PubMed and Embase searches used a combination of indexing and free-text terms and were restricted to English-language and Japanese-language articles published after 1 January 1996 and up to 6 August 2021 (the date of the searches). J-STAGE and CiNii searches were performed for the same time period directly within the respective online platform. Japanese-language articles were included because faropenem is approved for treating infectious diseases in Japan. Further details for the PubMed and Embase searches are provided in Table S1 (available as Supplementary data at *JAC-AMR* Online). Following the database searches, the title and abstract of identified articles/abstracts were screened for relevance based on *a priori* inclusion and exclusion criteria (Figure 2). Articles/abstracts were retained for full-text review if they: (1) included patients with infections treated with faropenem; (2) reported an outcome of clinical response, microbiological response or combined therapeutic response, reported safety or reported resistance to faropenem and/or prevalence of carbapenem cross-resistance; and (3) were randomized controlled trials, observational studies, systematic literature reviews and/or meta-analyses or preclinical studies. Specific exclusion criteria were set for the patient population, interventions, study design and article type, as detailed in Figure 2. Retained articles/abstracts finally underwent full-text review to determine eligibility for inclusion in the final dataset.

Two reviewers independently screened titles and abstracts, reviewed the full-text articles and performed data extraction for the articles ultimately selected for inclusion in the literature review. Disagreement between the two reviewers on the inclusion of an article was resolved by a third independent reviewer. All stages of the review were performed using Covidence systematic review management web-based software.

## Results

### Summary of identified studies

**Figure 3.**
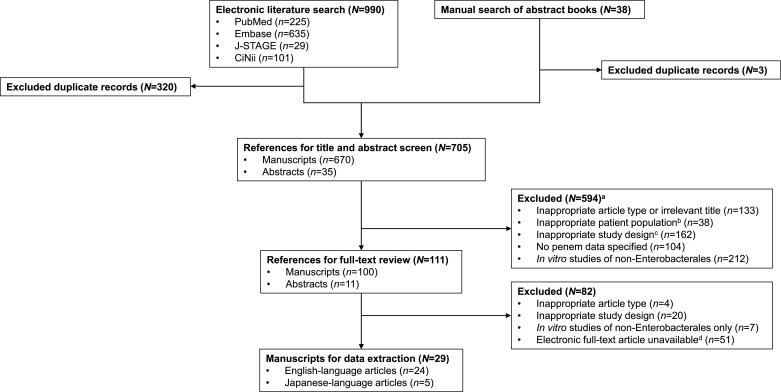
Literature search and systematic scoping literature review flow chart. ^a^Not mutually exclusive, some articles fell into >1 category. ^b^Included, but not limited to, healthy patients and patients with non-infectious diseases. ^c^Included, but not limited to, studies with combination oral penem treatment only and veterinary-focused studies. ^d^Article not available for download or inaccessible following special request.

The results of the literature search are shown in Figure 3. A total of 705 unique articles and abstracts published between 1 January 1996 and 6 August 2021 were identified from the database searches and were screened. Of these, 111 were selected for full-text review, with 29 publications ultimately satisfying all inclusion and exclusion criteria and subsequently included in the final dataset for this scoping systematic review. Twenty-four articles were English language and five were Japanese language, for which translations were obtained.

The 29 publications included 16 *in vitro* studies, 10 clinical trials, 2 studies reporting *in vitro and in vivo* data and 1 retrospective observational study (medical chart review). All studies investigated at least one oral penem, with faropenem the most common (*n *= 25), followed by sulopenem, ritipenem and MEN 10700 (*n *= 2 each). Across the 16 *in vitro* studies, the most investigated pathogens were *Escherichia coli* (*n *= 11; 68.8%), *Klebsiella pneumoniae* (*n *= 10; 62.5%), *Enterobacter* species (*n *= 6; 37.5%), *Citrobacter* species (*n *= 5; 31.3%) and *Morganella* species, *Serratia* species and *Proteus* species (all *n *= 4; 25.0%). Ten of the *in vitro* studies included at least one IV carbapenem as a comparator: imipenem (*n *= 10), meropenem (*n *= 7), doripenem (*n *= 6), biapenem (*n *= 4), panipenem (*n *= 4) and ertapenem (*n *= 3). One of the two *in vitro*/*in vivo* studies investigated MEN 10700 and ritipenem against *E. coli*, *Citrobacter* species and *Enterobacter* species, while the other investigated faropenem, L-036 and imipenem against *E. coli* and *K. pneumoniae*.

Of the 10 clinical trials, 7 were conducted in Japan, 1 in the USA and Canada, and 1 in multiple, mainly European, countries (one study did not report the country of origin). Eight trials included adult patients and two trials included paediatric patients. Five of the trials were randomized trials (the five Japanese-language articles were non-randomized trials) and six trials included a comparator: four included a different antibiotic as a comparator (cefuroxime *n *= 2, ciprofloxacin *n *= 1, levofloxacin *n *= 1) and two included an alternative faropenem regimen only. The sample size ranged from 15 to 1671 subjects, and the indications under investigation were uUTI (*n *= 6), respiratory infections (*n *= 3), chronic otitis media (*n *= 3), bacterial sinusitis (*n *= 2) and cUTI (*n *= 1). The retrospective chart review was conducted in Japan and included 10 adult patients with uUTI or cUTI who had been treated with faropenem.

### Evaluation of faropenem and sulopenem in clinical trials

Of the 10 clinical trials included in the review, 9^43–51^ investigated faropenem (1 in published abstract form only)^45^ and 1 investigated oral sulopenem (published abstract only).^52^ Details of the clinical trials, including summaries of the key efficacy and safety findings, are presented in Table 1. Below, we discuss notable themes that were identified during review of the trials.

**Table 1.** Details for the 10 clinical trials and single retrospective chart review

Author, year, country	Trial design, sample size	Indication	Population	Intervention and dosing regimen	Efficacy endpoints	Efficacy results^a^	Safety results^a^
Dunne *et al*.,^52^ 2020, not reported	Prospective randomized comparative trial *N *= 1671	uUTI	Adults (age not stated), 100% female	Sulopenem: twice daily, 5 days Ciprofloxacin: twice daily, 3 days	Overall therapeutic efficacy (defined as a combined clinical and microbiological response of success per subject)	Overall therapeutic response, test-of-cure visit (mITT) Combined population: *n *= 339/517 (65.6%) sulopenem versus *n *= 376/554 (67.9%) ciprofloxacin; difference −2.3% (95% CI: −7.9 to 3.3) Quinolone-non-susceptible population: *n *= 92/147 (62.6%) sulopenem versus *n *= 50/139 (36.0%) ciprofloxacin; difference 26.6% (95% CI: 15.1–37.4; *P *< 0.001) Quinolone-susceptible population: *n *= 247/370 (66.8%) sulopenem versus *n *= 326/415 (78.6%) ciprofloxacin; difference −11.8% (95% CI: −18.0 to −5.6) Overall therapeutic response, end-of-treatment visit (mITT) Combined population: *n *= 335/517 (64.8%) sulopenem versus *n *= 313/554 (56.5%) ciprofloxacin; difference 8.3% (95% CI: 2.4–14.1; *P *= 0.006) Quinolone-non-susceptible population: *n *= 95/147 (64.6%) sulopenem versus *n *= 42/139 (30.2%) ciprofloxacin; difference 34.4% (95% CI: 23.1–44.8; *P *< 0.001) Quinolone-susceptible population: *n *= 240/370 (64.9%) sulopenem versus *n *= 271/415 (65.3%) ciprofloxacin; difference −0.4% (95% CI: −7.1 to 6.2)	AEs Sulopenem: 24.8% ciprofloxacin: 13.9% Diarrhoea: 12.4% sulopenem versus 2.5% ciprofloxacin Drug-related AEs Sulopenem: 17.0% ciprofloxacin: 6.2% SAEs Similar between treatments
Suzuki *et al*.,^49^ 2016, Japan^b^	Prospective trial *N *= 15	Chronic otitis media	Adults (≥20 years), 40% female	Faropenem: 300 mg, once daily	Not reported	Not reported	AEs 6/15 (40.0%) Diarrhoea: *n *= 0/15 (0%) AEs possibly drug related *n *= 0/15 (0%) SAEs None
Hamasuna *et al*.,^44^ 2014, Japan	Prospective randomized comparative trial *N *= 200	uUTI	Adults (≥20 years), 100% female	Faropenem: 200 mg three times a day, 3 days Faropenem: 200 mg three times a day, 7 days	Microbiological efficacy (patients with bacterial eradication, persistence and replaced^c^) Clinical efficacy (success defined as the absence of symptoms)	Microbiological response, 5–9 days after treatment (ITT) Faropenem 3 days: *n *= 43/73 (58.9%) eradication, *n *= 15/73 (20.5%) persistence, *n *= 6/73 (8.2%) replaced Faropenem 7 days: *n *= 54/81 (66.7%) eradication, *n *= 5/81 (6.2%) persistence, *n *= 6/81 (7.4%) replaced 3 day versus 7 day: *P *= 0.048 Microbiological response, 4–6 weeks after treatment (ITT) Faropenem 3 days: *n *= 21/52 (40.4%) eradication, *n *= 3/52 (5.8%) relapsed, *n *= 2/52 (3.8%) reinfection Faropenem 7 days: *n *= 27/70 (38.6%) eradication, *n *= 4/70 (5.7%) relapsed, *n *= 3/70 (4.3%) reinfection 3 day versus 7 day: *P *= 0.839 Clinical response, 5–9 days after treatment (ITT) *n *= 56/73 (76.7%) faropenem 3 days versus *n *= 65/81 (80.2%) faropenem 7 days; *P *= 0.695 Clinical response, 4–6 weeks after treatment (ITT) *n *= 24/52 (46.2%) faropenem 3 days versus *n *= 35/70 (50.0%) faropenem 7 days; *P *= 0.717	AEs Total: *n *= 19/200 (9.5%) Faropenem 3 days: *n *= 10/97 (10.3%) faropenem 10 days: *n *= 9/103 (8.7%) Diarrhoea: *n *= 10/97 (10.3%) faropenem 3 days versus *n *= 5/103 (4.9%) faropenem 10 days
Hamasuna *et al*.,^45^ 2012, Japan	Prospective randomized comparative trial *N *= 200	uUTI	Adults (≥20 years), 100% female	Faropenem: 200 mg three times a day, 3 days Faropenem: 200 mg three times a day, 7 days	Microbiological efficacy outcome (patients with bacterial eradication, persistence and replaced^c^) Clinical efficacy (success not defined)	Microbiological response, 5–9 days after treatment Faropenem 3 days: *n *= 39/62 (62.9%) eradication, *n *= 15/62 (24.2%) persistence, *n *= 8/62 (12.9%) replaced Faropenem 7 days: *n *= 54/64 (84.4%) eradication, *n *= 5/64 (7.8%) persistence, *n *= 5/64 (7.8%) replaced 3 day versus 7 day: *P *= 0.018 Clinical response, 5–9 days after treatment *n *= 57/69 (82.6%) faropenem 3 days versus *n *= 66/71 (93.0%) faropenem 7 days; *P *= 0.061	Not reported
Yokota *et al*.,^51^ 2008, Japan^b^	Prospective trial *N *= 112	RTI, otitis media or uUTI	Paediatric (<16 years), 49% female	Faropenem: 15–30 mg/kg/day, 3–8 days	Clinical efficacy [defined as ratings of ‘Markedly effective’ and ‘Effective’ based on changes in subjective symptoms and objective findings (not defined)] Microbiological efficacy (success was defined as patients with bacterial eradication)	Clinical response, by disease (PP) Total: *n *= 91/100 (91.0%) Upper RTI: *n *= 63/70 (90.0%) Bronchitis: *n *= 6/7 (85.7%) Otitis media: *n *= 16/17 (94.1%) UTI: *n *= 6/6 (100%) Clinical response, by pathogen (PP) Total: *n *= 134/152 (88.2%) *Streptococcus pyogenes*: *n *= 42/47 (89.4%) *Streptococcus pneumoniae*: *n *= 25/27 (92.6%) *Haemophilus influenzae*: *n* = 43/50 (86.0%) *Moraxella catarrhalis*: *n* = 18/22 (81.8%) *E. coli*: *n* = 6/6 (100%) Microbiological response (PP) Total: *n *= 74/108 (68.5%) *S. pyogenes*: *n *= 36/40 (90.0%) *S. pneumoniae*: *n *= 13/16 (81.3%) *H. influenzae*: *n *= 13/33 (39.4%) *M. catarrhalis*: *n *= 6/13 (46.2%) *E. coli*: *n *= 6/6 (100%)	AEs *n *= 14/112 (12.5%) Diarrhoea: *n *= 14/112 (12.5%)
Upchurch *et al*.,^50^ 2006, USA, Canada	Prospective randomized comparative trial *N *= 1099	Acute bacterial sinusitis	Adults (≥18 years), 56% female	Faropenem: 300 mg twice daily, 7 days Faropenem: 300 mg twice daily, 10 days Cefuroxime: 250 mg twice daily, 7 days	Clinical efficacy (success defined as resolution or improvement of signs and symptoms and no requirement for further antimicrobial therapy)	Clinical response, 7–21 days after treatment (ITT) *n *= 262/366 (71.6%) faropenem 7 days versus *n *= 255/363 (70.2%) faropenem 10 days versus *n *= 250/370 (67.6%) cefuroxime 95% CI for difference from cefuroxime: faropenem 7 days: −2.7 to 10.5; faropenem 10 days: −3.9 to 9.5 Clinical response, 28–38 days after treatment (ITT) *n *= 237/366 (64.8%) faropenem 7 days versus *n *= 230/363 (63.4%) faropenem 10 days versus *n *= 222/370 (60.0%) cefuroxime	AEs Faropenem 7 days: *n *= 141/366 (38.5%) faropenem 10 days: *n *= 124/363 (34.2%) cefuroxime: 150/370 (40.5%) Gastrointestinal: 17% faropenem 7 days versus 14% faropenem 10 days versus 18% cefuroxime Drug-related AEs Faropenem 7 days: *n *= 81/366 (22.1%) faropenem 10 days: *n *= 73/363 (20.1%) cefuroxime: *n *= 69/370 (18.6%) Diarrhoea: 8% faropenem 7 days versus 4% faropenem 10 days versus 4% cefuroxime SAEs Faropenem 7 days: *n *= 1/366 (0.3%) faropenem 10 days: *n *= 2/363 (0.6%) cefuroxime: *n *= 6/370 (1.6%) (all drug unrelated)
Siegert *et al*.,^48^ 2003, France, Germany, Greece, Israel, Lithuania, Spain, Sweden	Prospective randomized comparative trial *N *= 561	Acute bacterial sinusitis	Adults (≥18 years), 57% female	Faropenem: 300 mg twice daily, 7 days Cefuroxime: 250 mg twice daily, 7 days	Clinical efficacy (success defined as disappearance of signs and symptoms, or significant improvement and not further therapy) Microbiological efficacy (success defined as patients with bacterial eradication or presumed eradication)	Clinical response, 7–16 days after treatment (PP) *n *= 203/228 (89.0%) faropenem versus *n *= 198/224 (88.4%) cefuroxime; 95% CI: −5.2 to 6.4 Clinical response, 28–35 days after treatment^d^ (PP) *n *= 188/203 (92.6%) faropenem versus *n *= 188/198 (95.0%) cefuroxime; 95% CI: −5.8 to 1.0 Microbiological response, 7–16 days after treatment (PP) *n *= 65/71 (91.5%) faropenem versus *n *= 59/65 (90.8%) cefuroxime; 95% CI: −9.2 to 9.5 Microbiological response, 7–16 days after treatment, by pathogen (PP) *M. catarrhalis*: *n *= 6/6 (100%) faropenem versus *n *= 5/6 (83.3%) cefuroxime *S. pneumoniae*: *n *= 36/37 (97.3%) faropenem versus *n *= 26/27 (96.3%) cefuroxime *Staphylococcus aureus*: *n *= 33/38 (86.8%) faropenem versus *n *= 38/42 (90.5%) cefuroxime *H. influenzae*: *n *= 17/20 (85%) faropenem versus *n *= 19/21 (90.5%) cefuroxime	AEs Total: *n *= 95/547 (17.4%) faropenem: *n *= 46/274 (16.8%) cefuroxime: *n *= 49/273 (17.9%) Digestive: *n *= 15/274 (5.5%) faropenem versus *n *= 18/273 (6.6%) cefuroxime Drug-related AEs Faropenem: *n *= 26/274 (9.5%) cefuroxime: *n *= 28/273 (10.3%) Diarrhoea: *n *= 6/274 (2.2%) faropenem versus *n *= 8/273 (2.9%) cefuroxime
Muratani *et al*.,^46^ 2002, Japan^b^	Prospective comparative trial *N *= 90	cUTI	Adults (≥16 years), 20% female	Faropenem: 300 mg three times a day, 7 days Levofloxacin: 100 mg three times a day, 7 days	Clinical efficacy (overall response, defined as a complete or partial response based on change in bacteriuria and pyuria) Microbiological efficacy (success defined as patients with bacterial eradication)	Clinical response, 7 days after treatment (PP) All UTI: *n *= 29/32 (90.6%) faropenem versus *n *= 23/28 (82.1%) levofloxacin; *P *= NS Polymicrobial infection: *n *= 9/11 (81.8%) faropenem versus *n *= 8/10 (80.0%) levofloxacin; *P *= NS Microbiological response, 7 days after treatment (PP) Bacterial eradication: *n *= 40/46 (87.0%) faropenem versus *n *= 33/40 (82.5%) levofloxacin; *P *= NS Bacterial persistence: *n *= 5/32 (15.6%) faropenem versus *n *= 6/28 (21.4%) levofloxacin Bacterial strains newly appearing: *n *= 5/32 (15.6%) faropenem versus *n *= 5/28 (17.9%) levofloxacin Microbiological response, by pathogen (PP) Gram-positive bacteria: *n *= 21/24 (87.5%) faropenem versus *n *= 12/16 (75.0%) levofloxacin MRSA: *n *= 0/1 (0%) faropenem versus *n *= 1/1 (100%) levofloxacin *Staphylococcus epidermis*: *n *= 3/3 (100%) faropenem versus *n *= 1/1 (100%) levofloxacin *Staphylococcus saprophyticus: n *= 1/1 (100%) faropenem *Staphylococcus* sp.: *n *= 4/6 (66.7%) faropenem versus *n *= 4/6 (66.7%) levofloxacin *Streptococcus agalactiae*: *n *= 2/2 (100%) faropenem versus *n *= 1/1 (100%) levofloxacin *Streptococcus bovis*: *n *= 1/1 (100%) faropenem versus *n *= 1/1 (100%) levofloxacin *Streptococcus* sp.: *n *= 3/3 (100%) faropenem versus *n *= 2/2 (100%) levofloxacin *Aerococcus* sp.: *n *= 0/1 (0%) levofloxacin *Enterococcus faecalis*: *n *= 7/7 (100%) faropenem versus *n *= 1/2 (50%) levofloxacin *Enterococcus gallinarum*: *n *= 1/1 (100%) levofloxacin Enterobacteriaceae: *n *= 15/18 (83.3%) faropenem versus *n *= 19/21 (90.5%) levofloxacin *Citrobacter braakii*: *n *= 1/1 (100%) faropenem *Citrobacter koseri*: *n *= 3/3 (100%) levofloxacin *Enterobacter aerogenes*: *n *= 1/1 (100%) levofloxacin *Enterobacter cloacae*: *n *= 2/2 (100%) faropenem *E. coli*: *n *= 9/11 (81.8%) faropenem versus *n *= 11/13 (84.6%) levofloxacin *Escherichia hermannii*: *n *= 1/1 (100%) levofloxacin *K. pneumoniae*: *n *= 2/2 (100%) faropenem versus *n *= 2/2 (100%) levofloxacin *Proteus mirabilis*: *n *= 1/1 (100%) levofloxacin *M. morganii*: *n *= 0/1 (0%) faropenem Other Gram-negative rods: *n *= 1/1 (100%) faropenem Glucose non-fermentable Gram-negative rods: *n *= 4/4 (100%) faropenem versus *n *= 2/3 (66.7%) levofloxacin *P. aeruginosa*: *n *= 0/1 (0%) levofloxacin *Pseudomonas fluorescens*: *n *= 1/1 (100%) levofloxacin *Alcaligenes faecalis*: *n *= 1/1 (100%) faropenem *Acinetobacter baumannii*: *n *= 1/1 (100%) faropenem *Acinetobacter lwoffii*: *n *= 1/1 (100%) faropenem *Stenotrophomonas maltophilia*: *n* = 1/1 (100%) faropenem versus *n* = 1/1 (100%) levofloxacin	AEs Total: *n *= 2/84 (2.4%) faropenem: *n *= 2/52 (3.8%) levofloxacin: *n *= 0/32 (0%) Diarrhoea: *n *= 2/52 (3.8%) faropenem versus *n *= 0/32 (0%) levofloxacin AEs possibly drug related Faropenem: *n *= 2/52 (3.8%)
Shiba *et al*.,^47^ 2002, Japan^b^	Prospective trial *N *= 17	RTI or uUTI	Adults (≥65 years), 41% female	Faropenem: 150 mg three times a day, 4–8 days	Not reported	Not reported	AEs *n *= 5/17 (29.4%) Diarrhoea: *n *= 2/17 (5.9%) AEs possibly drug related *n *= 3/17 (17.6%)
Fujii *et al*.,^43^ 1997, Japan^b^	Prospective trial *N *= 628	RTI, uUTI, otitis media	Paediatric (<16 years), 45% female	Faropenem: 3–5–10 mg/kg three times a day, 3–14 days	Clinical efficacy [ratings of ‘Excellent’ and ‘Good’ based on improvement of subjective and objective findings (not defined)] Microbiological efficacy (success defined as number of bacterial strains eradicated)	Clinical response, by diagnosis (PP) Total: *n *= 456/494 (92.3%) Patients with an isolate All respiratory infections: 90.5%–100% uUTI: *n *= 41/41 (100%) Otitis media: *n *= 15/21 (71.4%) Patients without an isolate All respiratory infections: 87.5%–100% uUTI: *n *= 10/10 (100%) Otitis media: *n *= 10/14 (71.4%) Clinical response, by midpoint dose (PP) 3 mg/kg three times a day: *n *= 14/16 (87.5%) 5 mg/kg three times a day: *n *= 213/228 (93.4%) 7.5 mg/kg three times a day: *n *= 128/139 (92.1%) 10 mg/kg three times a day: *n *= 92/102 (90.2%) Clinical response, by pathogen (PP) *S. aureus*: *n *= 55/65 (84.6%) *S. epidermidis*: *n *= 2/2 (100%) CoNS: *n *= 4/4 (100%) *S. pyogenes*: *n *= 64/67 (96.6%) *S. pneumoniae*: *n *= 23/25 (92.0%) Group A β-*Streptococcus*: *n *= 6/7 (85.7%) *E. faecalis*: *n *= 3/3 (100%) *M. catarrhalis*: *n *= 5/6 (83.3%) *E. coli*: *n *= 32/32 (100%) *Salmonella enteritidis*: *n *= 2/2 (100%) *S. typhimurium*: *n *= 1/1 (100%) *Salmonella* spp.: *n *= 2/2 (100%) *K. pneumoniae*: *n *= 1/1 (100%) *S. marcescens*: *n *= 1/1 (100%) *H. influenzae*: *n *= 32/34 (94.1%) *Haemophilus parainfluenzae*: *n *= 5/6 (83.3%) *Bordetella pertussis*: *n *= 3/3 (100%) *Campylobacter jejuni: n *= 2/2 (100%) Glucose non-fermenting Gram-negative rods: *n *= 1/1 (100%) Microbiological response (PP) Total: *n *= 250/303 (82.5%) Gram-positive: *n *= 162/188 (86.2%) Gram-negative: *n *= 88/115 (76.5%) Microbiological response, by pathogen (PP) *S. aureus*: *n *= 57/54 (77.0%) *S. epidermidis*: *n *= 2/2 (100%) CoNS: *n *= 4/4 (100%) *S. pyogenes*: *n *= 64/68 (94.1%) *S. pneumoniae*: *n *= 23/28 (82.1%) *Streptococcus sanguis*: *n *= 1/1 (100%) Group A β*-Streptococcus*: *n *= 4/4 (100%) Group G S*treptococcus*: *n *= 1/1 (100%) *E. faecalis*: *n *= 6/6 (100%) *M. catarrhalis*: *n *= 6/10 (60.0%) *E. coli*: *n *= 29/31 (93.5%) *Citrobacter freundii*: *n *= 1/1 (100%) *S. typhimurium*: *n *= 1/1 (100%) *S. enteritidi*s: *n *= 0/2 (0%) *Salmonella* spp.: *n *= 2/2 (100%) *K. pneumoniae*: *n *= 1/1 (100%) *S. marcescens*: *n *= 1/1 (100%) *P. mirabilis*: *n *= 1/1 (100%) *H. influenzae*: *n *= 37/51 (72.5%) *H. parainfluenzae*: *n *= 5/9 (55.6%) *B. pertussis*: *n *= 2/2 (100%) *C. jejuni*: *n *= 1/2 (50.0%) Glucose non-fermenting Gram-negative rods: *n *= 1/1 (100%)	AEs *n *= 36/548 (6.6%) Diarrhoea: *n *= 32/548 (5.8%) SAEs None
Fujino *et al*.,^54^ 2017, Japan	Retrospective chart review *N *= 10	uUTI or cUTI	Adults (24–86 years), 80% female	Faropenem: 200 mg three times a day, 7 days	Clinical efficacy (success defined as the resolution of all symptoms with no pyuria)	Clinical response *n *= 9/10 (90.0%) Recurrence^d^ *n *= 3/9 (33.3%)	Not reported

AE, adverse event; mITT, modified ITT; NS, not significant; RTI, respiratory tract infection; SAE, serious adverse event.

a Unless otherwise indicated, data are the number (%) of patients.

b Japanese-language article, for which full-text translation to English was obtained.

c The meaning of the term ‘replaced’ is not defined in the source article.

d Based on the number of patients who showed an initial clinical response as the denominator.

#### Oral penem dosing regimens varied broadly

There was distinct variation in the faropenem regimens used across the studies. This variation comprised differences in the dose, dosing frequency and duration of the regimen, and was in part dependent on the indication studied. In patients with uUTI, faropenem was administered as 200 mg three times a day for 3 or 7 days in two studies,^44,45^ as 150 mg three times a day for 4–8 days in another study^47^ and as 15–30 mg/kg/day for 3–8 days in a fourth study (Table 1).^51^ The single study of oral sulopenem also evaluated use in patients with uUTI and the drug was administered as 500 mg twice daily for 5 days.^52^ One study examined the efficacy of faropenem for treating cUTI and used a regimen of 300 mg three times a day for 7 days.^46^ The treatment of bacterial sinusitis was evaluated in two randomized studies; the regimen in each study was 300 mg twice daily either for 7 or 10 days.^48,50^ Patients with other indications such as respiratory tract infections^43,47,51^ and chronic otitis media^49,51^ were included in some studies, again with varied faropenem regimens (Table 1). Three studies included patients with different indications and used varied faropenem regimens within and between indications.^43,47,51^

#### Oral penem efficacy may be related to underlying antibiotic resistance

It seemed the clinical and/or microbiological response to oral penem treatment versus alternative antibiotics varied depending on the resistance mechanisms present in the causative bacteria. In the study by Dunne *et al*.^52^ investigating oral sulopenem for the treatment of uUTI, sulopenem was superior to ciprofloxacin in patients with a uUTI caused by a quinolone-non-susceptible pathogen [overall response of combined clinical and microbiological success: 62.6% versus 36.0%, difference 26.6% (95% CI: 1.51–37.4); *P *< 0.001], but was inferior when the causative pathogen was quinolone susceptible [overall response: 66.8% versus 78.6%, difference −11.8% (95% CI: −18.0 to −5.6)] (Table 1). In another study of patients with uUTI, Hamasuna *et al*.^44^ showed efficacy of faropenem at 5–9 days post-treatment based on both microbiological response (58.9%–66.7%) and clinical response (76.7%–80.2%). The authors reported high susceptibility (MIC ≤ 2 mg/L) to faropenem, including among *E. coli* strains resistant to levofloxacin (*n *= 11) and ESBL-producing strains resistant to cefcapene (*n *= 4), although the number of such strains was small.

#### Persistence and recurrence of UTI after treatment with faropenem

Two of the trials reported evidence of UTI persistence and/or recurrence after initial faropenem treatment success. In a randomized clinical trial of patients with uUTI receiving faropenem for 3 or 7 days, Hamasuna *et al*.^44^ reported bacterial persistence 5–9 days after treatment in 20.5% (3 day treatment) and 6.2% (7 day treatment) of patients (Table 1). Details of the resistant strains responsible for these persistent infections were not reported. Approximately 10% of the patients with initial treatment success (bacterial eradication) subsequently had uUTI recurrence (relapse or reinfection) at the 4–6 week follow-up (9.6% after 3 day treatment; 10.0% after 7 day treatment), although the responsible bacterial strains were not reported. Muratani *et al*.^46^ reported bacterial strains that persisted or newly appeared following treatment with faropenem for 7 days in a prospective trial of patients with cUTI. Seven days after treatment, six bacterial strains [in 5/32 (15.6%) patients] persisted—two strains each of *E. coli* and methicillin-resistant CoNS (MRCNS), and one strain each of *Morganella morganii* and MRSA; bacteriuria remained at ≥10^3^ cfu/mL for *M. morganii*, MRSA and one strain of MRCNS.^46^ This observation is perhaps not unexpected as faropenem is not likely to have activity against strains such as MRSA and *Morganella.* Also at 7 days post-treatment, eight bacterial strains, including *Serratia marcescens, Pseudomonas aeruginosa* and Gram-positive species such as MRSA, were newly isolated from five patients; six of the eight strains were reported to be highly resistant to faropenem based on susceptibility data (MIC ≥ 256 mg/L for *P. aeruginosa*, MRSA and *Enterococcus avium*; 32 mg/L for *S. marcescens* and MRSA; and 16 mg/L for *P. aeruginosa*). Characteristics of the five patients with the persistent bacterial strains and the four patients with the faropenem-resistant strains were not reported.

#### Oral penem treatment was associated with diarrhoea adverse events (AEs)

AEs following treatment with faropenem (sulopenem in one trial) were reported for all except one trial (Table 1). The incidence of AEs ranged from 3.8% (*n* = 2/84) in one study of patients with cUTI^46^ to 40.0% (*n *= 6/15) in a study of patients undergoing surgery for otitis media.^49^ The AEs most commonly reported were gastrointestinal/digestive, with mild-to-moderate diarrhoea reported in all but one of the studies.^43,44,46–48,50–52^ Diarrhoea is known to be a common side effect of antibiotic treatment.^53^ Where evaluated, the incidence of AEs and drug-related AEs was largely comparable between faropenem and comparator (i.e. cefuroxime or levofloxacin);^46,48,50^ in contrast, oral sulopenem for the treatment of uUTI was associated with more AEs (24.8% versus 13.9%) and drug-related AEs (17.0% versus 6.2%) than ciprofloxacin, largely attributable to a higher incidence of self-limited diarrhoea (12.4% versus 2.5%).^52^

#### Retrospective data on faropenem treatment and recurrent UTI

In addition to the clinical trials discussed, a retrospective observational study also reported clinical evidence of UTI recurrence following faropenem treatment. Fujino *et al*.^54^ conducted a retrospective review of medical chart data from patients treated with faropenem (standard regimen of 200 mg three times a day for 7 days) for a uUTI or cUTI caused by ESBL-producing *E. coli*. Faropenem resulted in a clinical cure (resolution of all symptoms with no pyuria) in 9 out of 10 patients (the clinical outcome was unknown in 1 patient who was admitted after changing faropenem to administration of sulfamethoxazole/trimethoprim; Table 1); however, three patients reportedly experienced a recurrent UTI that occurred 3–12 months after initial treatment completion that was caused by ESBL-producing *E. coli* (susceptibility results for faropenem were not reported for these recurrent infections).

### In vitro studies of faropenem and other oral penems

Included in the review were 16 *in vitro* studies, of which 4 were national antimicrobial susceptibility surveillance studies. Two additional *in vitro* studies also reported *in vivo* data. Details and key findings for the 18 studies are provided in Table 2. Discussed below are notable themes from review of these studies.

**Table 2.** Details for the *in vitro* studies and *in vitro* surveillance studies

Author, year, country	Sample source and size^a^	Indication	Penems and carbapenems	Enterobacterales species evaluated and results
*In vitro* studies				
Dawoud *et al*.,^63^ 2020, India	Clinical isolates *N *= 210	NR	Faropenem Imipenem	Resistance to faropenem and imipenem, respectively *E. coli* (*n *= 44): 2.3% and 4.5% *K. pneumoniae* (*n *= 34): 0% and 9.6%
Gandra *et al*.,^66^ 2020, USA	US CDC isolate bank *N *= 4	NR	Faropenem Imipenem Meropenem Doripenem Ertapenem	*In vitro* development of resistance to faropenem, change in MIC (mg/L) 1 to 64 within 10 days for two isolates, and 2 to 64 within 7 days for two isolates MIC range (mg/L) for imipenem; meropenem; doripenem; ertapenem ESBL-producing *E. coli*: 1–4; 2 to >8; 2 to >4; 8 to >8 Non-ESBL-producing *E. coli*: 1; ≤0.5; ≤0.5; ≤0.25
Karlowsky *et al*.,^60^ 2019, Canada	Clinical isolates *N *= 539	NR	Sulopenem	MIC range (mg/L) *E. coli*: 0.015–0.12 MIC_50_ (mg/L) *E. coli:* 0.03 MIC_90_ (mg/L) *E. coli*: 0.03
Nakamura *et al*.,^64^ 2014, Japan	Clinical isolates *N *= 210	NR	Faropenem	Rate of resistance (%) to faropenem^b^ *E. coli*: 0.6% (CLSI and EUCAST); 4.6% (JSC cystitis); 31.6% (JSC pyelonephritis) *K. pneumoniae*: 2.7% (CLSI and EUCAST); 5.4% (JSC cystitis); 37.8% (JSC pyelonephritis) MIC_50_ (mg/L) EBSL-producing *E. coli:* 1 EBSL-producing *K. pneumoniae*: 1 MIC_90_ (mg/L) EBSL-producing *E. coli*: 2 EBSL-producing *K. pneumoniae*: 2
Hu *et al*.,^68^ 2014, USA	Clinical isolates *N *= 135	NR	Faropenem Imipenem Meropenem Ertapenem Tebipenem	Mean (range) inhibition zone diameter (mm) for faropenem; imipenem; meropenem; ertapenem; tebipenem *K. pneumoniae* carbapenemase positive: *E. coli*: 6 (6); 16 (12–20); 15 (10–21); 13 (6–20); 16 (8–22) *K. pneumoniae*: 6 (6); 11 (6–22); 9 (6–20); 8 (6–16); 10 (6–21) *Enterobacter* spp.: 6 (6); 15 (7–23); 14 (7–20); 12 (6–17); 16 (7–22) *K. pneumoniae* carbapenemase negative: *E. coli*: 19 (10–24); 26 (22–30); 26 (25–30); 26 (22–30); 28 (26–32) *K. pneumoniae*: 19 (12–23); 26 (23–30); 26 (19–30); 26 (13–28); 28 (20–30) *Enterobacter* spp.: 16.5 (10–22); 23 (19–25); 25.5 (16–28); 24.5 (14–28); 27 (19–29) Zone diameter of 6 mm, for faropenem as a predictor of carbapenemase activity: 100% sensitivity and 100% specificity
Day *et al*.,^67^ 2013, International	Clinical isolates *N *= 453 (*N *= 248 part 1, *N *= 205 part 2)	NR	Faropenem Imipenem Meropenem Doripenem Ertapenem	Enterobacterales included in disc susceptibility testing: *E. coli*, *Klebsiella* spp., *Enterobacter* spp., *Citrobacter* spp., *P. mirabilis*, *Providencia rettgeri* and *Salmonella* spp. (all β-lactamase-producing) Mean (range) inhibition zone diameter (mm) for faropenem; imipenem; meropenem; doripenem; ertapenem CPE: 6 (6–12); 17 (6–24); 14 (6–28); 15 (6–26); 10 (6–28) Non-CPE: 19 (6–30); 27 (16–36); 28 (12–36); 28 (13–35); 25 (6–36) Zone diameter of 6 mm for faropenem as a predictor of carbapenemase activity Part 1: 99% sensitivity and 94% specificity Part 2: 98% sensitivity and 87% specificity
Mushtaq *et al*.,^56^ 2007, UK	Clinical isolates *N *= 847	NR	Faropenem Imipenem	MIC range (mg/L) for faropenem and imipenem, respectively *E. coli* CTX-M ESBL: 0.5–8 and 0.06–1 *E. coli* non-CTX-M ESBL: 0.25–8 and 0.06–0.5 *E. coli* AmpC: 0.5–16 and 0.06–0.5 *Klebsiella* spp. CTX-M ESBL: 0.12–16 and 0.12–2 *Klebsiella* spp. non-CTX-M ESBL: 0.12–16 and 0.12–2 *Klebsiella oxytoca* hyperproducing K1 enzyme: 0.25–1 and 0.12–0.5 *Enterobacter* spp. CTX-M ESBL: 1–8 and 0.12–1 *Enterobacter* spp. non-CTX-M ESBL: 1–16 and 0.06–1 *Enterobacter* spp. AmpC-derepressed: 0.5–16 and 0.12–2 *Citrobacter* spp. CTX-M ESBL: 2 and 0.25–0.5 *Citrobacter* spp. non-CTX-M ESBL: 0.5–1 and 0.12–1 *Citrobacter* spp. AmpC-derepressed: 0.5–8 and 0.25–1 *Serratia* spp. non-CTX-M ESBL: 1–2 and 0.25–0.5 *Serratia* spp. AmpC-derepressed: 4–16 and 0.12–2 *M. morganii*. AmpC-derepressed: 0.5–16 and 1–4 (All cephalosporin resistant)
Piddock *et al*.,^57^ 2003, UK	Clinical isolates *N *= 170	NR	Faropenem Imipenem	MIC range (mg/L) for faropenem and imipenem, respectively Ciprofloxacin-resistant *E. coli*: <0.06–0.5 and 0.125–0.125 MIC_50_ (mg/L) for faropenem and imipenem, respectively *S. pneumoniae:* <0.06 and <0.008 *S. aureus*: <0.006 and 1 *Enterococcus* spp.^b^: 16 and 64 *E. coli*: 0.125 and 0.125 *S. typhimurium*: 0.125 and 0.25 *P. aeruginosa*: 64 and 1 *Bacteroides fragilis*: 0.5 and <0.125 *C. jejuni*: 0.25 and 0.12 MIC_90_ (mg/L) for faropenem and imipenem, respectively *S. pneumoniae:* 0.5 and 0.25 *S. aureus*: 16 and 4 *Enterococcus* spp.^c^: >32 and 128 *E. coli*: 0.25 and 0.125 *S. typhimurium*: 0.25 and 0.5 *P. aeruginosa*: 128 and 8 *B. fragilis*: 8 and 4 *C. jejuni*: 0.25 and 0.25
Miyazaki *et al*.,^58^ 2001, Japan	Clinical isolates *N *= 483	NR	L-036 Faropenem Imipenem	MIC range (mg/L) for L-036; faropenem; imipenem *E. coli*: ≤0.063; 0.125–2; ≤0.063–0.25 *K. pneumoniae*: ≤0.063–0.5; 0.25–4; 0.125–0.5 MIC_50_ (mg/L) for L-036; faropenem; imipenem *E. coli:* ≤0.063; 0.5; 0.125 *K. pneumoniae*: ≤0.063; 0.5; 0.25 MIC_90_ (mg/L), for L-036, faropenem, imipenem *E. coli*: ≤0.063; 0.5; 0.125 *K. pneumoniae*: ≤0.063; 0.5; 0.25 MIC (mg/mL) *in vivo* in mice, for L-084 (prodrug) and faropenem, respectively Penicillin-susceptible *S. pneumoniae* TUH39: 0.0005 and 0.001
Okuda *et al*.,^59^ 2000, Japan	Standard strains and clinical isolates *N *= 758	NR	Faropenem DU-6681a R-95867	MIC range (mg/L) for faropenem; DU-6681a; R-95856 *E. coli*: 0.12–2; ≤0.008–0.03; ≤0.008–0.06 *K. pneumoniae*: 0.25 to >128; ≤0.008–2; 0.015–4 *S. marcescens*: 2–128; 0.03–32; 0.06–32 *Enterobacter* spp.: 0.25–8; 0.015–0.25; 0.03–2 *C. freundii*: 1–4; 0.015–0.06; 0.03–0.5 *Proteus* spp.: 0.5–8; 0.03–0.5; 0.03–0.5 *M. morganii*: 1–8; 0.06–0.5; 0.06–1 *Shigella* spp.: 0.25–1; ≤0.008–0.03; 0.015–0.06 MIC_50_ (mg/L) for faropenem; DU-6881a;R-95856 *E. coli:* 1; ≤0.008; 0.015 *K. pneumoniae*: 0.5; 0.015; 0.015 *S*. *marcescens*: 8; 0.12; 0.5 *Enterobacter* spp.: 2–4; 0.03; 0.25 *C*. *freundii*: 2; 0.03; 0.12 *Proteus* spp.: 2; 0.06–0.12; 0.12 *M. morganii*: 2; 0.25; 0.5 MIC_90_ (mg/L) for faropenem; DU-6881a; R-95856 *E. coli:* 1; 0.015; 0.03 *K. pneumoniae*: 1; 0.06; 0.06 *S*. *marcescens*: 32; 0.25; 2 *Enterobacter* spp.: 4–8; 0.12; 0.5–2 *C*. *freundii*: 4; 0.06, 0.5 *Proteus* spp.: 4–8; 0.25–0.5; 0.25–0.5 *M. morganii*: 8, 0.25, 1
Arcamone *et al*.,^65^ 2000, Italy	NR	NR	MEN 10700 Ritipenem	General findings Citing other sources, MEN 10700 showed good to excellent activity against most Enterobacterales, and had a spectrum of activity similar to ritipenem and faropenem, but with higher activity on *Enterobacter* and *Citrobacter* species Stability (after 190 min incubation at 37°C), MEN 10700 and ritipenem, respectively 68% unchanged and 35% unchanged AUC (mg/L × min) *in vivo* in rats, for MEN 11505 (prodrug) and ritipenem acoxil (prodrug), respectively 283.2 and 229.0 Relative bioavailability *in vivo* in rats, for MEN 11505 (prodrug) and ritipenem acoxil (prodrug), respectively 43% and 23%
Woodcock *et al*.,^55^ 1997, UK	Standard strains and clinical isolates *N *= 726	NR	Faropenem	MIC range (mg/L) *E. coli*: 0.06–8 *Klebsiella* spp.: 0.06–8 *Proteus* spp.: 0.25–4 *M. morganii*: 0.06–4 *Serratia* spp.: 1–128 *Enterobacter* spp.: 0.5–16 *Citrobacter* spp.: 0.25–4 *Salmonella* spp.: 0.5 *Shigella* spp.: 0.25–0.5 *Providencia stuartii*: 0.06–4 MIC_50_ (mg/L) *E. coli*: 0.5 *Klebsiella* spp.: 0.5 *Proteus* spp.: 1 *M. morganii*: 1 *Serratia* spp.: 2 *Enterobacter* spp.: 2 *Citrobacter* spp.: 0.5 *Salmonella* spp.: 0.5 *Shigella* spp.: 0.5 *P. stuartii*: 1 MIC_90_ (mg/L) E. coli: 1 Klebsiella spp.: 2 Proteus spp.: 2–4 M. morganii: 2 Serratia spp.: 32 Enterobacter spp.: 4 Citrobacter spp.: 4 Salmonella spp.: 0.5 Shigella spp.: 0.5 P. stuartii: 2
Hamilton-Miller *et al*.,^61^ 1997, UK	Clinical isolates *N *= 539	NR	MEN 10700 Ritipenem	MIC range (mg/L) for MEN 10 700 and ritipenem, respectively *E. coli*: 0.25–1 and 1 *K. pneumoniae*: 0.5–1 and 1–2 *Enterobacter* spp.: 0.5–4 and 1–32 *C. freundii*: 0.5–4 and 4–32 *P. mirabilis*: 4–8 and 4–32 *M. morganii*: 1–16 and 4–16 *S. marcescens*: 0.5–8 and 4–16 *Providencia* spp.: 1–16 and 1–32 MIC_50_ (mg/L) for MEN 10 700 and ritipenem, respectively *E. coli*: 0.5 and 1 *K. pneumoniae*: 1 and 1 *Enterobacter* spp.: 2 and 16 *C. freundii*: 1 and 8 *P. mirabilis*: 8 and 4 *M. morganii*: 2 and 8 *S. marcescens*: 2 and 8 *Providencia* spp.: 2–8 and 4–16 MIC_90_ (mg/L) for MEN 10 700 and ritipenem, respectively *E. coli*: 0.5 and 1 *K. pneumoniae*: 1 and 1 *Enterobacter* spp.: 2 and 32 *C. freundii*: 2 and 16 *P. mirabilis*: 8 and 4 *M. morganii*: 16 and 16 *S. marcescens*: 4 and 8 *Providencia* spp.: 8–16 and 16–32
Frean *et al*.,^62^ 1996, Namibia	Clinical isolates *N *= 100	Plague	Faropenem	MIC range (mg/L) *Y. pestis*: <0.03–0.5 MIC_50_ (mg/L) *Y. pestis:* 0.25 MIC_90_ (mg/L) *Y. pestis*: 0.5
*In vitro* surveillance studies				
Yanagihara *et al*.,^72^ 2020, Japan	Clinical isolates *N *= 1062	Respiratory infections	Faropenem Imipenem Meropenem Doripenem Biapenem Panipenem	MIC range (mg/L) for faropenem; imipenem; meropenem; doripenem; biapenem; panipenem *K. pneumoniae*: 0.25–8; 0.125–2; ≤0.06–0.25; ≤0.06–0.5; ≤0.06–4; 0.125–1 MIC_50_ (mg/L) *K. pneumoniae:* 0.5; 0.25; ≤0.06; ≤0.06; 0.25; 0.25 MIC_90_ (mg/L) *K. pneumoniae*: 1; 1; ≤0.06; 0.125; 1; 0.5
Watanabe *et al*.,^71^ 2012, Japan	Clinical isolates *N *= 684	Respiratory infections	Faropenem Imipenem Meropenem Doripenem Biapenem Panipenem	MIC range (mg/L) for faropenem; imipenem; meropenem; doripenem; biapenem; panipenem *K. pneumoniae*: 0.125–32; ≤0.06–1; ≤0.06–0.125; ≤0.06–0.125; ≤0.06–1; ≤0.06–0.5 MIC_50_ (mg/L) *K. pneumoniae:* 0.5; 0.125; ≤0.06; ≤0.06; 0.125; 0.125 MIC_90_ (mg/L) *K. pneumoniae*: 0.5; 0.5; ≤0.06; ≤0.06; 0.5; 0.5
Niki *et al*.,^69^ 2011, Japan	Clinical isolates *N *= 1097	Respiratory infections	Faropenem Imipenem Meropenem Doripenem Biapenem Panipenem	MIC range (mg/L) for faropenem; imipenem; meropenem; doripenem; biapenem; panipenem *K. pneumoniae*: 0.125–8; ≤0.06–2; ≤0.06–0.25; ≤0.06–0.25; ≤0.06–1; ≤0.06–2 MIC_50_ (mg/L) *K. pneumoniae:* 0.25; 0.25; ≤0.06; ≤0.06; 0.125; 0.25 MIC_90_ (mg/L) *K. pneumoniae*: 1; 1; ≤0.06; ≤0.06; 0.25; 0.5
Niki *et al*.,^70^ 2009, Japan	Clinical isolates *N *= 1178	Respiratory infections	Faropenem Imipenem Meropenem Doripenem Biapenem Panipenem	MIC range (mg/L) for faropenem; imipenem; meropenem; doripenem; biapenem; panipenem *K. pneumoniae*: 0.25–4; ≤0.06–1; ≤0.06–0.125; ≤0.06–0.25; ≤0.06–1; ≤0.06–1 MIC_50_ (mg/L) *K. pneumoniae:* 0.5; 0.25; ≤0.06; ≤0.06; 0.25; 0.25 MIC_90_ (mg/L) *K. pneumoniae*: 1; 0.5; ≤0.06; ≤0.06; 0.5; 0.5

AmpC, AmpC β-lactamase; CPE, carbapenemase-producing Enterobacterales; CTX-M, CTX type M variant; JSC, Japanese Society of Chemotherapy; NR, not reported.

a Sample size is the total sample and includes both Enterobacterales and non-Enterobacterales strains/isolates.^b^Based on susceptibility criteria for ertapenem of 2 mg/L (CLSI and EUCAST) and for meropenem of 2 mg/L for cystitis and 1 mg/L for pyelonephritis (JSC), as a substitute for faropenem.

c Includes *E. faecalis* and *Enterococcus faecium*.

#### Oral penem MIC data

A notable, and perhaps anticipated, theme was the variation in reported MIC values across studies for faropenem and other oral penems against isolates of different Enterobacterales pathogens. Taking *E. coli* as an exemplar pathogen associated with uUTI, faropenem was reported to have an MIC range of 0.06–8 mg/L and an MIC_90_ of 0.5 mg/L in a 1997 UK study,^55^ while two other UK studies, one in 2003 (sample collection date not specified) and one in 2007 (samples collected in 2004), reported MIC ranges of <0.06–0.5 mg/L and 0.25–16 mg/L, respectively; the 2003 study also reported an MIC_90_ of 0.125 mg/L (Table 2).^56,57^ In Japan, the MIC range for faropenem against *E. coli* was 0.125–2 mg/L in a study with samples from 1995–1997 (the MIC_90_ value was 0.5 mg/L)^58^ and 0.12–2 mg/L in a study from 2000 (the MIC_90_ value was 1 mg/L).^59^ For sulopenem, the MIC range and MIC_90_ against *E. coli* was 0.015–0.12 mg/L and 0.03 mg/L, respectively, in a Canadian study using samples from 2014–2016.^60^ MIC ranges and MIC_90_ values of 1 mg/L and 1 mg/L, respectively, were reported for ritipenem and 0.25–1 mg/L and 0.5 mg/L, respectively, were reported for MEN 10700 in a 1997 study from the UK.^61^

The MIC range for faropenem against other uUTI-related pathogens was broader (Table 2): for *Klebsiella* species, 0.06–8 mg/L (the MIC_90_ was 1 mg/L) and 0.12–16 mg/L in two UK studies in 1997 and 2004, respectively,^55,56^ and 0.25–4 mg/L (the MIC_90_ was 0.5 mg/L) and 0.25 to >128 mg/L in two Japanese studies in 1995–1997 and 2000, respectively;^58,59^ for *Citrobacter* species, 0.25–4 mg/L (the MIC_90_ was 4 mg/L) and 0.5–8 mg/L in the 1997 and 2004 UK studies^55,56^ and 1–4 mg/L (the MIC_90_ was 4 mg/L) in a 2000 study from Japan;^59^ and for *Serratia* species, 1–128 mg/L (the MIC_90_ was 32 mg/L) and 1–16 mg/L in the 1997 and 2004 UK studies^55,56^ and 2–128 mg/L (the MIC_90_ was 32 mg/L) in the 2000 study from Japan.^59^ Faropenem MIC ranges for other Enterobacterales have also been reported, including against *Enterobacter* species—0.5–16 mg/L in the 1997 and 2004 studies from the UK (the MIC_90_ was 4 mg/L in the 1997 study)^55,56^ and 0.25–8 mg/L (the MIC_90_ was 4–8 mg/L) in the 2000 study from Japan^59^—and an MIC range of <0.03–0.5 mg/L (the MIC_90_ was 0.5 mg/L) against *Yersinia pestis* in a study from Namibia analysing samples collected between 1982 and 1991.^62^ Ritipenem and MEN 10700 each had an MIC range against *K. pneumoniae* broadly comparable to their reported range against *E. coli* (1997 UK study); the MIC_90_ was 1 mg/L for both therapies.^61^ Of note, three oral carbapenems, DU-6681a, R-95867 and L-036, had MIC ranges against *E. coli* [≤0.008–0.03 mg/L (MIC_90_: 0.015 mg/L);  ≤ 0.008–0.06 mg/L (MIC_90_: 0.03 mg/L);  ≤ 0.063 mg/L (MIC_90_: ≤ 0.063 mg/L)], *K. pneumoniae* [≤0.008–2.0 mg/L (MIC_90_: 0.06 mg/L); 0.015–4.0 mg/L (MIC_90_: 0.06 mg/L);  ≤ 0.063–0.5 mg/L (MIC_90_: ≤ 0.063 mg/L)] and *S. marcescens* [0.03–32 mg/L (MIC_90_: 0.25 mg/L); 0.06–32 mg/L (MIC_90_: 2 mg/L); not tested] that were markedly lower than those for faropenem, as reported by studies in 2000 and 2001 from Japan.^58,59^

#### Faropenem resistance among ESBL-producing strains and cross-resistance to carbapenems

One study conducted in India by Dawoud *et al*.^63^ examined susceptibility and rate of resistance among ESBL-producing *E. coli* and *K. pneumoniae* isolates to faropenem and imipenem. Susceptibility testing was conducted according to CLSI 2008 guidelines, but details of how susceptibility and resistance were determined was not reported. While most ESBL-producing isolates were susceptible to both antibiotics, there was evidence of resistance to faropenem in 2.3% of ESBL-producing *E. coli* isolates but not in ESBL-producing *K. pneumoniae*; resistance to imipenem was higher [4.5% (*E. coli*) and 9.6% (*K. pneumoniae*)] (Table 2). Similarly, using isolates collected in Japan from 2000–2009, Nakamura *et al*.^64^ earlier reported high susceptibility to faropenem among ESBL-producing *E. coli* (99.4%) and *K. pneumoniae* (97.3%) according to CLSI 2010 and EUCAST guidelines; as these guidelines do not have MIC breakpoints for faropenem, the findings were based on the susceptibility MIC breakpoint of 2 mg/L for ertapenem (same drug type with the most similar biokinetics). When the authors used JSC criteria based on susceptibility breakpoints for meropenem (2 mg/L for cystitis and 1 mg/L for pyelonephritis) as a substitute for faropenem, evidence of resistance to faropenem was greater: 4.6% and 31.6% of ESBL-producing *E. coli* and 5.4% and 37.8% of ESBL-producing *K. pneumoniae*, were not susceptible (resistant) according to JSC criteria for uUTI and pyelonephritis, respectively (Table 2).^64^ Another penem, MEN 10700, reportedly demonstrated activity against Enterobacterales species including ESBL-producing strains, but resistance data were not reported.^65^ Plasma levels and relative bioavailability of MEN 10700 were higher than for ritipenem after oral administration in rats of the prodrugs MEN 11505 and ritipenem acoxil.^65^

Potential resistance to faropenem has also been demonstrated in antibiotic-resistant ESBL-producing strains. In a study investigating faropenem activity in cephalosporin-resistant Enterobacterales, Mushtaq *et al*.^56^ reported that approximately 5% of ESBL-producing *E. coli* and *Klebsiella* species collected from 16 UK centres in 2004 had a faropenem MIC of >2–8 or >2–16 mg/L, respectively, suggestive of some resistance. ESBL-producing *Enterobacter* species showed greater evidence of potential resistance, with an MIC of >2–16 mg/L for 59% of isolates.^56^ In these experiments, imipenem showed MIC ranges two-to-three doubling dilutions lower than those of faropenem. Separately, a study with *E. coli* strains resistant to ciprofloxacin did not show evidence of resistance to faropenem (MIC range <0.06–0.5 mg/L) or to imipenem (MIC range 0.125–0.125 mg/L) (whether strains were ESBL-producing was not reported) (Table 2).^57^

A recent *in vitro* study by Gandra *et al*.^66^ examined the possibility that faropenem resistance might cause cross-resistance to carbapenems. Using *E. coli* isolates from the US CDC antibiotic resistance isolate bank, the authors demonstrated that the development of resistance to faropenem through serial passage resulted in the development of cross-resistance to carbapenem antibiotics.^66^ This was restricted to ESBL-producing isolates, and reduced carbapenem susceptibility was noted in three CTX-M-15-producing isolates exhibiting faropenem resistance. Whereas the MIC before the development of faropenem resistance was ≤0.5 mg/L for all tested carbapenems, this increased to ≥8 mg/L for ertapenem,  ≥ 2 mg/L for doripenem and meropenem and ≥1 mg/L for imipenem (Table 2).^66^ The use of faropenem for *in vitro* disc susceptibility testing has been shown to be a predictive (98%–100% sensitivity, 87%–100% specificity) screening test for carbapenemase-producing Enterobacterales;^67,68^ Day *et al*.^63^ utilized Enterobacteriaceae from diverse international sources to demonstrate this. This test might thus be used in a manner analogous to ESBL testing to predict carbapenemase production with high sensitivity and specificity.

It is important to consider the explosion of CTX-M-type ESBLs circa 2007–2010 when evaluating studies of faropenem activity among ESBL-producing strains. If CTX-M-type enzymes are driving faropenem resistance, as some observations suggest,^66^ then studies conducted prior to 2007 may underestimate the level of resistance that might be observed today. Results from the cited studies conducted by Mushtaq *et al*.,^56^ Piddock *et al*.^57^ and Arcamone *et al*.^65^ must therefore be interpreted with this possibility in mind.

#### In vitro surveillance studies

Four of the *in vitro* studies were nationwide surveillance studies conducted in Japan.^69–72^ Each study reported the *in vitro* antimicrobial susceptibility of bacterial respiratory pathogens from clinical isolates collected in 2007, 2008, 2009 or 2016. Over 40 antibacterial agents, including faropenem and carbapenems, and between 684 and 1178 bacterial strains, of which *K. pneumoniae* was the only Enterobacterales pathogen, were evaluated in each study. Against *K. pneumoniae* isolates, the reported MIC range for faropenem was 0.25–4 mg/L (*n *= 126 isolates) in 2007,^70^ 0.125–8 mg/L (*n *= 122) in 2008,^69^ 0.125–32 mg/L (*n *= 78) in 2009^71^ and 0.25–8 mg/L (*n *= 134) in 2016.^72^ For each year, the reported faropenem MIC range was broader and had a higher upper value than the range for each of the tested carbapenems (imipenem, panipenem, meropenem, biapenem and doripenem) (Table 2).

## Discussion

Carbapenems are typically ‘last resort’ antibiotics for the treatment of severe and life-threatening bacterial infections and those caused by MDR pathogens.^9^ However, the role of oral penem antibiotics is unclear. Oral penem antibiotics (e.g. faropenem) have been developed but currently are approved only in Japan and India. As such, fewer data are available compared with antibiotics used more globally. There is a need for better understanding of the public health impact of faropenem and other oral penems as there is concern for cross-resistance to carbapenems. This scoping literature review was conducted to begin to address this by exploring the impact of faropenem and other oral penems on the treatment of Enterobacterales infections and evaluating the evidence for faropenem resistance and potential for cross-resistance to carbapenem antibiotics.

We found a paucity of evidence in the published literature that describes the use of faropenem in treating infectious diseases. Of the 29 articles that we identified, only 4 were randomized clinical trials with faropenem, only 2 of which included a different antibiotic as a comparator. Most of the studies we identified (*n *= 18) were preclinical *in vitro* investigations. As such, the current clinical evidence base for faropenem is very limited, more so in the context of individual indications. Nevertheless, we did identify several notable themes from the available clinical studies.

This included broad variation in faropenem dosing regimens within and between indications (150, 200 or 300 mg, once daily, twice daily or three times a day, from 3 to 10 days; as well as 15–30 mg/kg per day for 3–14 days).^43–51,54^ None of the studies provided details on recommended dosing of faropenem. Joint recommendations from the Japanese Association for Infectious Disease (JAID) and JSC advocate faropenem 200 mg three times a day for 7 days to treat uUTI, but only where ESBL-producing bacteria are present or suspected.^31^ This regimen was used in three of six studies that included patients with uUTI;^44,45,54^ all six studies were conducted in Japan and predate the JAID/JSC recommendations. A faropenem regimen of 200–300 mg twice daily is recommended by Medindia for treating genitourinary infections;^33^ however, Indian national treatment guidelines do not list faropenem for genitourinary infections.^32^ Another notable finding was the potential dependence of treatment efficacy on the underlying resistance mechanisms of the causative pathogen. In one randomized trial (available as a published abstract only), the efficacy of sulopenem versus ciprofloxacin for treating uUTI reportedly differed dependent on susceptibility of the causative pathogen to quinolones,^52^ while another trial that included only faropenem showed efficacy for uUTI caused by *E. coli* resistant to different fluoroquinolones or to cephalosporins.^44^ No direct comparisons can be made between these trials given differences in study design regarding the investigational drug and dosing regimen, patient population, comparator and study endpoints. Also of particular interest, we found evidence from several studies of UTI persistence and recurrence following initial, efficacious treatment with faropenem.^44,46,54^ While the proportion with recurrence was low (10%) in the clinical trial,^44^ and the sample size small (*n *= 10; 3/9 with clinical cure had a recurrence) in the retrospective chart review study,^54^ these data illustrate the potential for repeated infections, including from the same causative pathogen^54^ or from different pathogens that show resistance to faropenem.^46^

The primary purpose of this scoping review was to answer three research questions on the use of faropenem in the treatment of infectious diseases. Ultimately, the limited body of relevant data we identified prohibits satisfactory answers to these questions. In the first question we sought to determine the prevalence of faropenem resistance among Enterobacterales. To our knowledge, specific breakpoints for faropenem susceptibility and resistance have yet to be defined.^73^ Data from included studies suggest that resistance to faropenem exists in a range of bacterial pathogens. Many studies reported faropenem MIC ranges against various bacterial species, although did not specifically state resistance rates.^43,44,55–59,62,69–72^ In some cases, such as strains of *E. coli*, *K. pneumoniae* and *S. marcescens*, the upper value of the reported MIC range suggested some resistance to faropenem. One *in vitro* study did report resistance rates, reporting that resistance to faropenem was detectable in some Enterobacterales pathogens—2.3% of ESBL-producing *E. coli* isolates were resistant to faropenem, but none of the tested ESBL-producing *K. pneumoniae* isolates were.^63^ Another study also indicated very low resistance of these pathogens to faropenem, although evidence for resistance was somewhat greater according to different susceptibility criteria.^64^ Separately, data indicate that resistance to faropenem is likely through a mechanism distinct from that of fluoroquinolone resistance.^57^ Interestingly, a recent *in vitro* study investigating genetic determinants of resistance reported that faropenem treatment of *K. pneumoniae* clinical isolates with and without a β-lactamase gene resulted in the isolation of resistant mutants to faropenem for all isolates, suggesting that β-lactamases may not be required for the development of faropenem resistance in some cases.^74^ While the available evidence overall suggests that faropenem resistance exists at a low level in some Enterobacterales, dedicated efforts are needed to robustly evaluate resistance to this antibiotic across bacterial species, indications and populations.

The use (and misuse) of antibiotics contributes to the development of antimicrobial resistance.^75^ Accordingly, treatment of bacterial infections with faropenem may facilitate the emergence of resistance to this antibiotic, the focus of our second research question. While available data were limited, there was evidence of faropenem resistance in a clinical trial from Japan in patients with cUTI who received a 7 day regimen of faropenem.^46^ Seven days after treatment, six strains in five patients persisted, with bacteriuria of ≥10^3^ cfu/mL for three strains (MRSA, MRCNS and *M. morganii*) that would not be considered susceptible to treatment with faropenem. Moreover, eight bacterial strains were newly detected in 5/32 patients 7 days after treatment completion and six of these strains were reported, based on MIC data, to be highly resistant to faropenem; notably, some of these isolates were not the same strains that had caused the initial infections. Bacterial persistence (6%–20% of patients) suggestive of resistance to faropenem treatment was also reported separately in a trial of patients with uUTI in Japan,^44^ although biofilm formation^76^ in the bladder or recurrent dysbiosis^77^ of the urinary tract might also explain this observation. It should be noted that neither of these two trials nor any of the other identified clinical trials included faropenem resistance as a trial endpoint. To put these data for faropenem into context, resistance to fluoroquinolones among isolates of *E.coli*, an exemplar pathogen associated with uUTI, is well established, but with broad global variation, ranging from <5% to ≥80% of isolates, and a range in Japan of 40% to <50% of isolates.^1^ Some data are also available for another oral penem, sulopenem. Dunne *et al*.^78^ compared oral sulopenem to oral ciprofloxacin in adult women with uUTI, reporting microbiological success (eradication of baseline pathogen to <1000 cfu/mL) in patients with ciprofloxacin-non-susceptible uropathogens of 74.1% with sulopenem and 49.6% with ciprofloxacin, i.e. bacterial persistence of 25.9% and 50.4%, respectively.

An *in vitro* study by Gandra *et al*.^66^ was the only study identified in our searches that was relevant to the third of our research questions on whether the development of resistance to faropenem may foster cross-resistance to carbapenem antibiotics. Among ESBL-producing *E. coli* isolates with CTX-M-15-type enzymes, the development of resistance to faropenem caused cross-resistance to multiple carbapenems, namely ertapenem, doripenem, meropenem and imipenem, to varying degrees.^66^ There was some evidence of loss of resistance to some of these carbapenems over serial passaging after 10 days in antibiotic-free media (as seen by reduced MIC values versus before passaging), dependent on the isolate examined. A reason for this observation might be the differential antibiotic permeability of the isolates due to differing degrees of porin expression and ESBL quantities; *ompC* expression and ESBL production were not quantified in the study. Of note, the non-ESBL-producing isolate, NSF4, which has no known genetic mechanism of resistance, did not develop cross-resistance to carbapenems. Mutations in the *envZ* and *gaIU* genes may have resulted in high-level faropenem resistance in this pan-susceptible isolate, but further molecular studies are needed to confirm this.

Beyond the study by Gandra *et al*.,^66^ recent *in vitro* investigations by Ma *et al*.^74^ also provide support for the potential of faropenem resistance to foster cross-resistance to carbapenems. The authors examined genetic determinants of resistance using clinical isolates of *K. pneumoniae*, finding that exposure to faropenem resulted in resistant mutants to faropenem, which in turn promote evolution of resistance to the carbapenem meropenem.^74^ In the presence of meropenem, the faropenem-resistant mutant exhibited elevated mutation frequencies and conjugation efficiencies relative to the parental strain (despite an unchanged MIC of meropenem), suggesting that faropenem treatment promotes the selection of mutations that may increase the likelihood of developing high-level resistance to meropenem. It is important to note that resistance in this study was defined as an increase in MIC for the resistant mutant relative to the parental strain of at least 2-fold (not relative to defined MIC breakpoints).

Overall, the data discussed provide preliminary evidence showing that resistance may develop following exposure to faropenem, and that resistance to this penem antibiotic may foster cross-resistance to carbapenems in certain bacterial strains. Whether penems other than faropenem cause cross-resistance to carbapenems has not, to our knowledge, been investigated; however, the structural similarities among penems and carbapenems does suggest this may occur. These data emphasize the importance of the appropriate use of faropenem and oral penems to treat severe infections and the use of more narrow-spectrum antibiotics to treat common infections in the community, such as uUTI, thus preserving penems for those who really need them. Addressing these issues will require concerted efforts to reverse the chronic lack of investment and insufficiency of current research and antimicrobial surveillance programmes within the community, a setting accounting for a substantial proportion of antibiotic prescribing.

This work has several limitations. We acknowledge that our search strategy, while broad in scope, may not have identified all relevant non-English published literature. To better identify relevant material for screening, we searched multiple electronic databases as well as the online proceedings of prominent infectious diseases congresses. In addition to searches of the PubMed and Embase databases, we also searched two Japanese databases, J-STAGE and CiNii, on the basis that faropenem has been approved in Japan. The output of our searches was also limited by the choice of search terms. However, we used a variety of indexing and free-text terms, some general, some more specific, to assemble a broader body of articles for screening. We also acknowledge that 51 articles passed initial screening for relevance but could not be reviewed due to unavailability of the electronic full-text article, which is a limitation. This was despite submitting special requests for full-text access. It is likely that 1 or more of these 51 articles reported data relevant to this review.

The dearth of clinical evidence describing the use of faropenem for treating infectious diseases emphasizes a pressing unmet need for well-designed, comparative, clinical trials of faropenem in patients with common indications such as uUTI. Such studies should include, as a point of importance, evaluation of the persistence of symptoms at the end of treatment, disease recurrence in patients initially treated successfully, asymptomatic intestinal colonization post-treatment and the emergence of faropenem resistance post-exposure. Whether the development of antimicrobial resistance to faropenem may ultimately foster cross-resistance to carbapenems (oral or IV) remains unanswered at this time, but preliminary evidence from one study included in this review suggests this might occur.^66^ It is a point that must be addressed with urgency through basic science and clinical research, given the essential role of carbapenems as the ‘last resort’ antibiotics for those patients with serious/life-threatening infections or an MDR infection.

In conclusion, the published literature currently has very limited clinical evidence describing the use of faropenem for the treatment of infectious diseases. Faropenem is approved in Japan and India, but no clinical trials in the latter country were identified, while those in Japan were restricted in their design. We were unable to satisfactorily answer the research questions on which this review was founded; these and other important questions thus remain. From a public health perspective, judicious use of faropenem within the community is essential to preserve its broad spectrum of activity for patients who really need it. Our findings illustrate a pressing need for investment not only in preclinical and clinical research, but also in dedicated surveillance monitoring within the community to comprehensively explore patterns of faropenem treatment and resistance, including carbapenem cross-resistance.

## Supplementary Material

dlac125_Supplementary_Data

## Writing assistance statement

Medical writing support for the literature search screening and data extraction was provided by Joanna Wilson, PhD, and Natasha Tracey, PhD, of Ashfield MedComms, an Inizio company. Medical writing support with manuscript development, grammatical editing, figure and table redraws and collating of author comments (under the guidance of the authors) was provided by Joanna Wilson, PhD, of Ashfield MedComms, an Inizio company, and was funded by GSK.

## Supplementary data

Table S1 is available as Supplementary data at *JAC-AMR* Online.
