# Supplementary material for: A systematic scoping review of faropenem and other oral penems: treatment of Enterobacterales infections, development of resistance and cross-resistance to carbapenems
Source: JAC Antimicrob Resist. 2022 Dec 22;4(6):dlac125. doi: 10.1093/jacamr/dlac125 (PMC9777757; doi:10.1093/jacamr/dlac125)
Supplement: dlac125_Supplementary_Data [file dlac125_supplementary_data.docx]

**Supplementary Table S1.** Indexing and free-text terms used in PubMed and Embase database searches

| **Search number** | **Query** | **Subject** |
| --- | --- | --- |
| 1 | "fropenem"[Supplementary Concept] OR "fropenem"[All Fields] OR "faropenem"[All Fields] OR "faropenem medoxomil"[All Fields] OR "faropenem daloxate"[All Fields] OR "SUN 5555"[Supplementary Concept] OR "SUN 5555"[All Fields] OR sun5555[All Fields] OR SY5555[All Fields] OR WY49605[All Fields] OR RU67655[All Fields] OR ALP201[All Fields] OR "BLA 857"[All Fields] OR "BLA-857"[All Fields] OR "YM 044"[All Fields] OR "SUN-5555"[All Fields] OR "WY 49605"[All Fields] OR "WY-49605"[All Fields] OR "ALP 201"[All Fields] OR "ALP-201"[All Fields] OR "SY 5555"[All Fields] OR "SY-5555"[All Fields] OR Farobact[All Fields] OR Farom[All Fields] OR Orapem[All Fields] OR A0026[All Fields] OR "SUN 208"[All Fields] OR SUN208[All Fields] OR "SUN-208"[All Fields] OR "SUN A0026"[All Fields] OR "SUN-A0026"[All Fields] OR "BAY 56-6854"[All Fields] OR "BAY56-6854"[All Fields] OR "BAY-56-6854"[All Fields] OR "BAY 566854"[All Fields] OR "BAY566854"[All Fields] OR "BAY-566854"[All Fields] OR ("penem"[All Fields] AND (oral[All Fields] OR orally[All Fields])) | Oral penem index and free-text terms |
| 2 | #1 AND eng[la] | Oral penem English language-only |
| 3 | #1 AND jpn[la] | Oral penem Japanese language-only |
| 4 | #2 AND ("1996/01/01"[Date - Publication]: "3000"[Date - Publication]) | Oral penem English language-only, publication after 1995 |
| 5 | #3 AND ("1996/01/01"[Date - Publication]: "3000"[Date - Publication]) | Oral penem Japanese language-only, publication after 1995 |
